# Identification of a Novel Antagonist of BRS-3 from Natural Products and Its Protective Effects Against H_2_O_2_-Induced Cardiomyocyte Injury

**DOI:** 10.3390/ijms26062745

**Published:** 2025-03-18

**Authors:** Jihong Lu, Lehao Wu, Jianzheng Zhu, Han Zhou, Mingzhu Fang, Hongshuo Liang, Miao Guo, Mo Chen, Yuhang Zhu, Jixia Wang, Hua Xiao, Yan Zhang

**Affiliations:** 1 Shanghai Frontiers Science Center of Drug Target Identification and Delivery, School of Pharmacy, Shanghai Jiao Tong University, Shanghai 200240, China; lujihong@sjtu.edu.cn (J.L.); wulehaogo@sjtu.edu.cn (L.W.); cris08@sjtu.edu.cn (M.F.); dlutlhs@163.com (H.L.); chen-mo@sjtu.edu.cn (M.C.); 2 State Key Laboratory of Microbial Metabolism, Joint International Research Laboratory Metabolic & Developmental Sciences, School of Life Sciences and Biotechnology, Shanghai Jiao Tong University, Shanghai 200240, China; zhujzh9@sjtu.edu.cn (J.Z.); guoniao@sjtu.edu.cn (M.G.); huaxiao@sjtu.edu.cn (H.X.); 3 Key Laboratory of Phytochemistry and Natural Medicines, Dalian Institute of Chemical Physics, Chinese Academy of Sciences, Dalian 116023, China; zhouhan418@dicp.ac.cn (H.Z.); jxwang@dicp.ac.cn (J.W.); 4 Zhiyuan College, Shanghai Jiao Tong University, Shanghai 200240, China; zhuyhang18119625017@sjtu.edu.cn

**Keywords:** BRS-3, orphan GPCR, licoisoflavone A, antagonist, cardiomyocyte

## Abstract

The identification of exogenous ligands from natural products is an alternative strategy to explore the unrevealed physiological functions of orphan G-protein-coupled receptors (GPCRs). In this study, we have successfully identified and pharmacologically characterized licoisoflavone A (LIA) as a novel selective antagonist of BRS-3, an orphan GPCR. Functional studies showed that pretreatment with LIA ameliorated hydrogen peroxide (H_2_O_2_)-induced cardiomyocyte injury. Furthermore, LIA pretreatment significantly restored the activities of malondialdehyde (MDA), superoxide dismutase (SOD), and catalase (CAT), as well as lactate dehydrogenase (LDH) levels, in H9c2 cells following H_2_O_2_ exposure. The protective effect of LIA was also evident in primary cardiomyocytes from rats and mice against H_2_O_2_-induced cell injury but was absent in primary cardiomyocytes derived from bombesin receptor subtype-3 knockout (*Brs3*^−/y^) mice, strongly confirming the mechanism of LIA’s action through BRS-3 antagonism. Proteomics studies further revealed that LIA exerted its protective effects via activating the integrin/ILK/AKT and ERK/MAPK signaling pathways. Complementary findings from Bantag-1, a well-recognized antagonist of BRS-3, in human embryonic kidney 293 mBRS-3 (HEK293-mBRS-3) stable cells and B16 cell lines, which demonstrated resistance to H_2_O_2_-induced damage, further supported the pivotal role of BRS-3 in oxidative stress-induced cell injury. Our study contributes to expanding our understanding of the potential pharmacological functions of BRS-3, unveiling previously unknown pharmacological functionality of this orphan receptor.

## 1. Introduction

G protein-coupled receptors (GPCRs), known as seven transmembrane receptors, are the therapeutic targets of approximately 40% of modern medicinal drugs [1]. Of all the validated GPCRs, 25% have no known endogenous ligands and are, thus, classified as orphan receptors [2]. The orphan receptor bombesin receptor subtype-3 (BRS-3, BB_3_) was identified based on its high degree of homology to neuromedin B receptor (NMBR, BB_1_) and the gastrin-releasing peptide receptor (GRPR, BB_2_), both of which are recognized as mammalian bombesin receptors [3].

BRS-3 couples with a guanine nucleotide-binding protein (G_q_), decomposing the 4,5-diphosphatidylinositol biphosphate (PIP2) into inositol triphosphate (IP3) and diacylglycerol. This process leads to an elevation of cytosolic calcium (Ca^2+^) and the subsequent activation of protein kinase C (PKC) [4]. Given its implications for energy homeostasis, feeding behavior, and insulin regulation [5], BRS-3 continues to attract interest in both academia and industry. With its broad expression in both central and peripheral tissues, the role of BRS-3 in various human diseases is gradually being elucidated [6]. However, the exploration of BRS-3’s physiological and pathological roles is hindered not only by the absence of endogenous ligands, but also by the scarcity of specific pharmacological tools. Currently, there are only a few exogenous ligands available, notably the agonist MK-5046 and the antagonist Bantag-1 [7], with antagonists being particularly scarce and in limited supply.

As part of our ongoing project to identify new ligands capable of interacting with BRS-3, this study has successfully unveiled a novel antagonist, licoisoflavone A (LIA), derived from natural products. LIA is a natural flavonoid and the main component of Glycyrrhiza [8]. LIA is also an effective ingredient in numerous traditional Chinese medicine prescriptions, notably the Tongmaiyangxin pill [9] and Baoyuan decoction [10]. Previous studies have shown the therapeutic efficacy of the Tongmaiyangxin pill and Baoyuan decoction in treating cardiovascular diseases, particularly myocardial injury [9,10]. LIA has also been reported to exhibit anti-myocardial hypertrophy activity [9]. We have, for the first time, demonstrated that LIA exerts a protective effect against H_2_O_2_-induced cardiomyocyte injury in a BRS-3-dependent manner. This study holds significant importance as it marks the novel pharmacological functionality of the orphan receptor BRS-3, paving the way for potential therapeutic applications.

## 2. Results

### 2.1. LIA Is a Novel Antagonist with a High Selectivity of BRS-3

**Figure 1:**
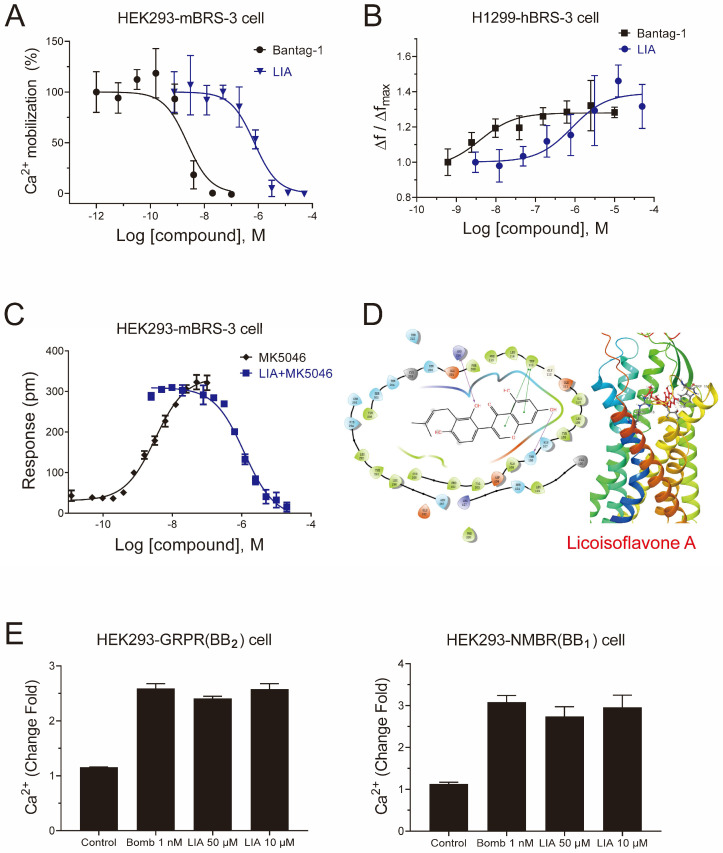
Pharmacological characterization of LIA as a novel antagonist of BRS-3. Dose–inhibition curves of LIA for 50 nM MK-5046-induced calcium mobilization in HEK293-mBRS-3 cells (**A**), IP-1 accumulation in H1299-hBRS-3 cell (**B**), and DMR responses in HEK293-mBRS-3 cells (**C**). The predicted binding model of LIA with human BRS-3 (**D**). The ability of LIA to reverse 1 nM Bomb-induced calcium mobilization in HEK293 expressing GRPR and NMBR cells (**E**). Data shown are means ± SD, *n* = 3. Dose–response curves were fitted using the log(agonist) vs. response using GraphPad Prism 8.0 software.

For G_q_-coupled GPCRs, intracellular Ca^2+^ signaling is initiated upon agonist binding by converting G_αq_-GDP to G_αq_-GTP, which triggers phospholipase C (PLC)-dependent IP3 generation and Ca^2+^ release. As shown in Figure 1A, LIA, as well as Bantag-1, a well-characterized BRS-3 antagonist, dose-dependently reversed 50 nM MK-5046-induced intracellular Ca^2+^ mobilization in HEK293-mBRS-3 stable cells. The IC_50_ value of LIA was 7.063 × 10^−7^ M (4.250 × 10^−7^ M to 1.167 × 10^−6^ M, 95% CI), while that of Bantag-1 was 2.316 × 10^−9^ M (1.301 × 10^−9^ M to 4.172 × 10^−9^ M, 95% CI).

To further verify the antagonistic effect of LIA, we examined the IP1 accumulation, i.e., the stable degradation product of IP3, in H1299-hBRS-3 stable cells. As shown in Figure 1B, the accumulation caused by 50 nM MK-5046 could be inhibited by LIA with an IC_50_ value of 8.410 × 10^−7^ M (1.564 × 10^−7^ M to 3.482 × 10^−6^ M, 95% CI). We also established the dynamic mass redistribution (DMR) assay in HEK293-mBRS-3 and found that LIA reversed the DMR response of 50 nM MK-5046 in a dose-dependent manner with an IC_50_ value of 1.185 × 10^−6^ M (8.387 × 10^−7^ M to 1.674 × 10^−6^ M, 95% CI) (Figure 1C).

In docking studies, the calculated binding energy was −53.38 kJ/mol and the docking score was −7.847, indicating the tight binding between LIA and the receptor. The amino acid residues Thr^106^, Arg^316^, and Trp^113^ contributed to the binding interactions. The predicted binding site Arg^316^ is the same as for the previously studied agonists [11,12]. The binding between LIA and BRS-3 was visualized in Figure 1D.

To assess the receptor subtype selectivity of LIA, HEK293 cells were transiently transfected with either BB_1_ and BB_2_. [D-Phe^6^, β-Ala^11^, Phe^13^, Nle^14^] Bombesin (6-14) (Bomb), a full agonist capable of activating both BB_1_ and BB_2_, substantially triggered Ca^2+^ mobilization. Notably, LIA, even at a concentration of 50 μM (Figure 1E), failed to reverse this Ca^2+^ mobilization, therefore highlighting the subtype selectivity of LIA.

Collectively, these results indicate that LIA, a natural product, is a novel selective antagonist of BRS-3.

### 2.2. LIA Protects Cardiomyocytes Against H_2_O_2_-Induced Injury in H9c2 Cells

**Figure 2:**
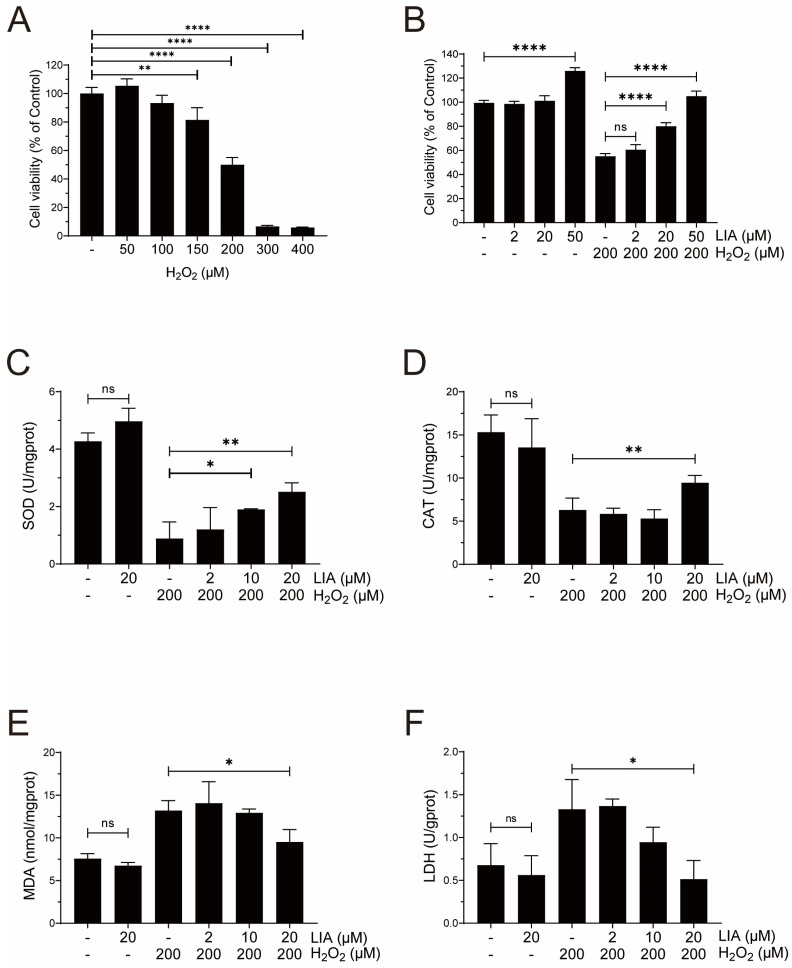
The protective effects of LIA on H_2_O_2_-induced injury in H9c2 cells. Cell viability at different concentrations of H_2_O_2_ (**A**). Protective effect of LIA on 200 μM H_2_O_2_-induced cell injury (**B**). Protective effect of LIA on the activities of SOD (**C**), CAT (**D**), and the levels of MDA (**E**) and LDH (**F**). The values are represented as means ± SD, *n* = 3. * *p* < 0.05, ** *p* < 0.01, **** *p* < 0.001. “ns” stands for “not significant”, indicating *p* > 0.05.

H9c2 cells were incubated with various concentrations of H_2_O_2_ for 24 h to mimic myocardial injury in vitro. H_2_O_2_ caused a dose-dependent decrease in cell viability (Figure 2A). It was found that when 200 μM H_2_O_2_ was used, the inhibition rate of H9c2 cells reached approximately 50%. Subsequently, 200 μM H_2_O_2_ was applied for modeling. As shown in Figure 2B, the H_2_O_2_-induced decrease in cell viability was significantly increased after 20 and 50 μM LIA treatment. Since 50 μM LIA was observed to affect cell viability (Figure 2B), 20 μM LIA was used as the highest concentration in the following experiments.

As shown in Figure 2C,D, exposure of H9c2 cells to H_2_O_2_ induced oxidative damage, and resulted in a marked decrease in superoxide dismutase (SOD) activities compared to the control group. These reductions were effectively reversed by LIA treatment. Furthermore, as depicted in Figure 2E,F, the levels of malondialdehyde (MDA) and lactate dehydrogenase (LDH) were increased by H_2_O_2_ stimulation; however, these increases were significantly counteracted following LIA treatment.

Taken together, the above results suggest that LIA confers a protective effect against H_2_O_2_-induced cellular damage in H9c2 cells.

### 2.3. Proteomics Analysis of the Effect of LIA in H9c2 Cells Induced by H_2_O_2_

**Figure 3:**
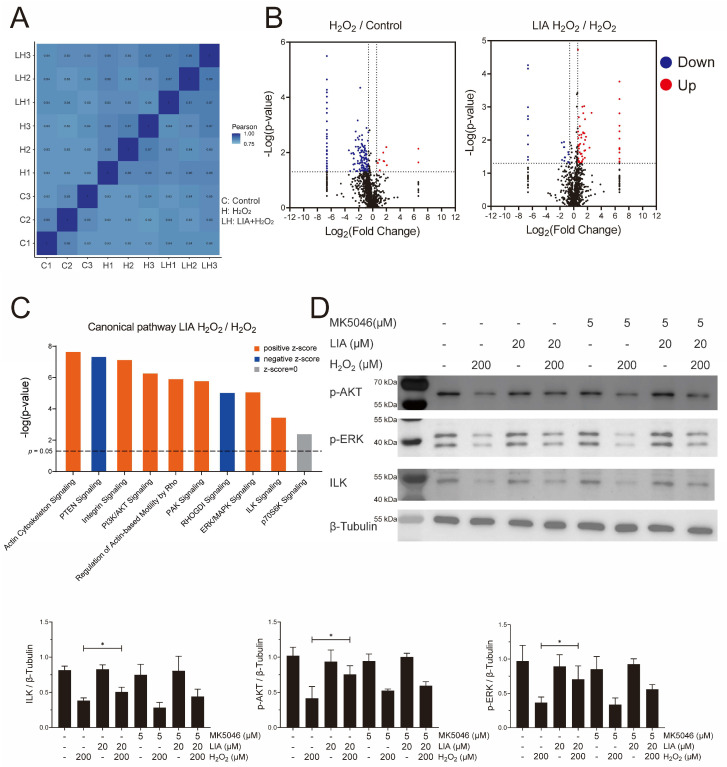
Proteomics analysis of the effect of LIA in H9c2 cells induced by H_2_O_2_. Pearson correlation (**A**) and Volcano plots (**B**). The red dots represent proteins that were upregulated (*p* < 0.05), and the blue dots represent proteins that were downregulated (*p* < 0.05). The black dots represent proteins with fold change < 1.5 or without significant changes (*p* > 0.05). “Canonical pathway” enrichment (**C**), the dash line represents *p* = 0.05. The effects of LIA on the protein expressions of ILK, *p*-P70S6K, *p*-ERK1/2, and *p*-AKT (Ser^473^) were detected by Western blot (**D**). The values are represented as means ± SD, *n* = 3. * *p* < 0.05.

Quantitative proteomics analysis was performed to further explore the mechanism of the protective effect of LIA in H9c2 cells. LC-MS/MS was used to analyze three biological repeats for each group (C: Control, H: H_2_O_2_, LH: LIA + H_2_O_2_), and the Pearson correlation of these raw data was calculated (Figure 3A). In total, 1853 proteins were identified. Volcano plots (Figure 3B) showed that 140 proteins were downregulated and 10 were upregulated after H_2_O_2_ stimulation. Moreover, compared with solvent control, LIA pretreatment resulted in 55 proteins being upregulated and 22 being downregulated.

To obtain a deep insight into LIA-induced signaling pathways, the canonical pathway enrichment was analyzed by IPA (Figure S1). Several major pathways, including integrin signaling, PI3K/AKT signaling, ERK/MAPK signaling, ILK signaling, and p70S6K signaling, were revealed (Figure 3C) based on their −log(*p*-value). Interestingly, these pathways are closely related to cardiac biology and pathophysiology. Therefore, we focused on these pathways during subsequent pathway verification.

Western blot analysis showed that in H9c2 cells, ILK, *p*-AKT (Ser^473^), and *p*-ERK1/2 were downregulated in the H_2_O_2_ group, although this downregulation was reversed upon LIA treatment (Figure 3D). However, treatment with LIA alone exhibited no significant impact on the levels of ILK, *p*-AKT (Ser^473^), and *p*-ERK1/2 levels in normal control cells. Furthermore, MK-5046 has the trend to diminish the protective effects of LIA against H_2_O_2_-induced cell damage. In addition, the phosphorylation of p70S6K1 at Thr^389^ showed no significant change with LIA treatment (Figure S3). These findings were in line with the proteomics analysis results.

### 2.4. LIA Protects H_2_O_2_-Induced H9c2 Cell Injury via ILK/AKT and ILK/ERK Signaling Pathways

**Figure 4:**
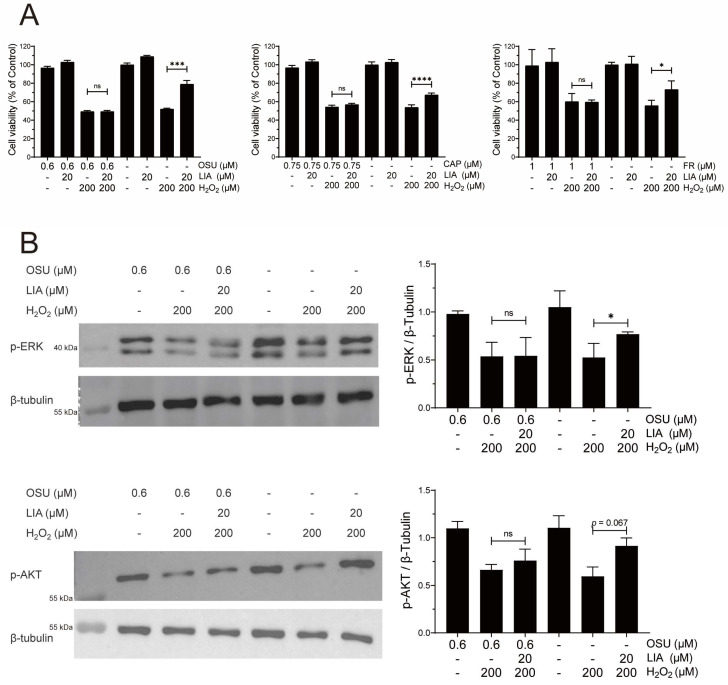
Roles of ILK/AKT and ILK/ERK signaling pathways in the protective effects of LIA against H_2_O_2_-induced H9c2 cell injury. Effects of LIA on cell viability in H9c2 cells pretreated with specific inhibitors (**A**). The protein expressions of *p*-ERK1/2 and *p*-AKT (Ser^473^) after treatment with ILK inhibitor (**B**). The values are represented as means ± SD, *n* = 3. * *p* < 0.05, *** *p* < 0.005, **** *p* < 0.001. “ns” stands for “not significant”, indicating *p* > 0.05.

The roles of ILK, PI3K/AKT, and ERK/MAPK signaling pathways in the protective effects of LIA were further investigated. H_2_O_2_-induced H9c2 cells were pretreated with specific inhibitors of ILK (OSU-T315, 0.6 μM), AKT (Capivasertib, 0.75 μM), and ERK1/2 (FR 180204, 1 μM), respectively, before LIA treatment. The results showed that the three inhibitors—OSU, CAP, and FR—each significantly diminished the protective effects of LIA, as illustrated in Figure 4A. Interestingly, OSU, an ILK inhibitor, was observed to counteract the LIA-induced elevation of *p*-AKT (Ser^473^) and *p*-ERK1/2, as shown in Figure 4B. This finding implies that ILK may be upstream in the PI3K/AKT and ERK/MAPK signaling cascades.

### 2.5. LIA Exerts Protective Effects Against H_2_O_2_-Induced Injury in Primary Rat and Mouse Cardiomyocytes

**Figure 5:**
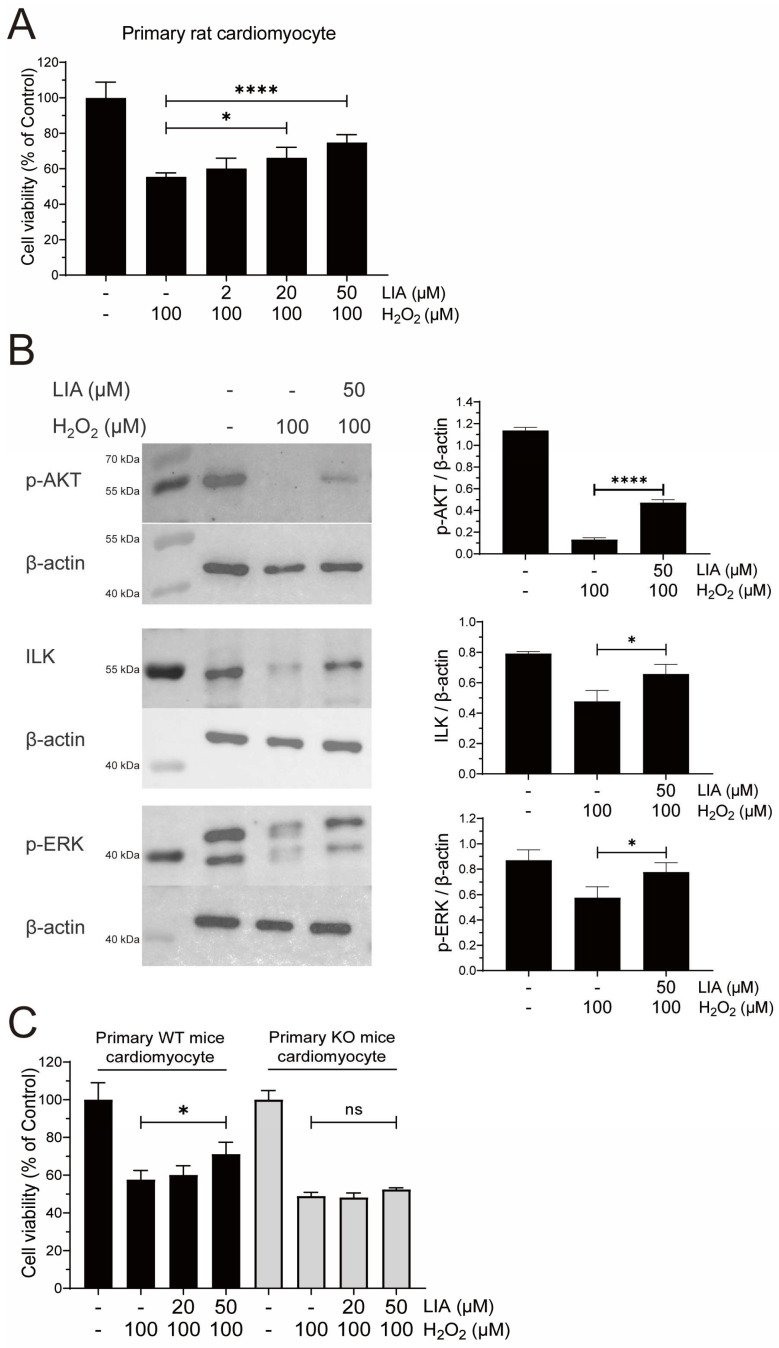
The protective effects of LIA against H_2_O_2_-induced injury in primary rat and mouse cardiomyocytes. The effect of LIA on cell viability in primary rat and mouse cardiomyocytes (**A**). LIA alters the protein expressions of ILK, *p*-P70S6K, *p*-ERK1/2, and *p*-AKT (Ser^473^) against H_2_O_2_-induced injury in primary rat cardiomyocytes (**B**). The comparative analysis of the effects of LIA on cell viability against H_2_O_2_-induced injury in primary cardiomyocytes from wild-type (WT) and *Brs3*^−/y^ (KO) mice (**C**). The values are represented as means ± SD, *n* = 6 for (**A**), *n* = 3 for (**B**), and *n* = 5 for (**C**). * *p* < 0.05, **** *p* < 0.001. “ns” stands for “not significant”, indicating *p* > 0.05.

To substantiate the protective effects of LIA against H_2_O_2_-induced cellular damage, we isolated and cultured primary cardiomyocytes from rats and mice. Due to the limited proliferative capacity of primary cardiomyocytes [13], a concentration of 50 μM LIA was chosen in the experiment to achieve a better signal. Notably, LIA exhibited pronounced protective effects in both primary rat and mouse cardiomyocytes compared to the solvent control group (Figure 5A).

Consistent with the observations in H9c2 cells, treatment with LIA effectively activated the ILK, AKT (Ser^473^), and ERK1/2 signaling pathways in primary rat cardiomyocytes subjected to H_2_O_2_-induced injury, as depicted in Figure 5B.

To further validate the target, we acquired primary cardiomyocytes from *Brs3*^−/y^ mice along with their littermates, for a comparative analysis. As shown in Figure 5C, our results demonstrated that 50 μM LIA specifically increased the viability of cardiomyocytes derived from wild-type mice, whereas it had no such effect on those from *Brs3*^−/y^ mice. This distinct response provides compelling evidence that the protective effect of LIA is mediated by its interaction with BRS-3.

### 2.6. BRS-3 Inhibition Regulates H_2_O_2_-Induced Cell Injury

Our findings demonstrate that LIA, a novel selective antagonist of BRS-3, effectively protects against H_2_O_2_-induced cardiomyocyte injury. This strongly suggests the role of BRS-3 in the pathogenesis of oxidative stress-induced cell injury. To validate our hypothesis, we performed quantitative real-time reverse transcription polymerase chain reaction (qRT-PCR) tests to detect and quantify the expression of BRS-3 in cardiomyocytes as shown in Figure S4A. The data confirm that BRS-3 is endogenously expressed in both H9c2 and rat primary cardiomyocytes. Furthermore, we assessed the phosphorylation of ERK in H9c2 cells in response to MK-5046, a well-characterized BRS-3 agonist. The results revealed that MK-5046 promoted ERK phosphorylation, while both Bantag-1 and LIA inhibited this agonist-induced phosphorylation, indicating that BRS-3 is not only expressed but also functional in H9c2 cells, as depicted in Figure S4B.

**Figure 6:**
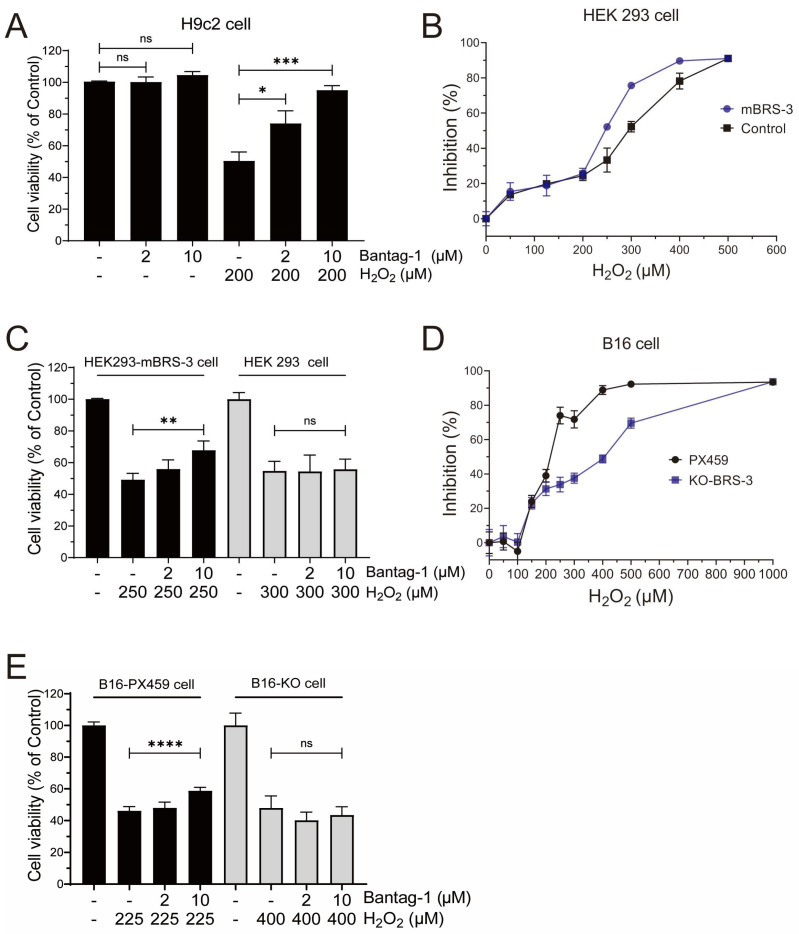
Validation of target for the BRS-3 regulation of H_2_O_2_-induced cell injury. Effect of Bantag-1 on cell viability in H9c2 cells (**A**). Cell viability in different concentrations of H_2_O_2_ in HEK293 and HEK293-mBRS-3 stable cells (**B**). Effect of Bantag-1 and LIA on cell viability against H_2_O_2_-induced injury in HEK293 and HEK293-mBRS-3 stable cells (**C**). Cell viability in different concentrations of H_2_O_2_ in B16 (B16-PX459) cells and B16-KO cells (**D**). Comparative analysis of the effects of Bantag-1 on cell viability against H_2_O_2_-induced injury in B16 cells and B16-KO cells (**E**). The values are represented as means ± SD, *n* = 3 for (**A**–**D**) and *n* = 4 for (**E**). * *p* < 0.05, ** *p* < 0.01, *** *p* < 0.005, **** *p* < 0.001. “ns” stands for “not significant”, indicating *p* > 0.05.

We then proceeded to investigate the impact of Bantag-1, a recognized antagonist of BRS-3 on H9c2 cells, instead of LIA. Interestingly, our observations revealed that Bantag-1 had no influence on cell viability in the control group; however, in the presence of H_2_O_2_, Bantag-1, but not MK-5046 (Figure S2), significantly increased cell viability (Figure 6A), demonstrating a protective effect similar to that of LIA.

To rule out the potential influence of cell line specificity and for target validation, we conducted a comparative analysis of the H_2_O_2_-induced response in both HEK293 and HEK293-mBRS-3 stable cells. Our findings indicated that 250 μM H_2_O_2_ reduced the cell viability of HEK293-mBRS-3 cells to 47.86%, whereas a higher concentration of 300 μM H_2_O_2_ was required to achieve a comparable reduction in HEK293 cells (Figure 6B). In addition, when pretreated with 10 μM Bantag-1, the cell viability of HEK293-mBRS-3 cells was markedly improved compared to the H_2_O_2_ model group (Figure 6C). However, such enhancements were absent in HEK293 cells.

We recently found that BRS-3 was endogenously expressed in B16 cells, the murine melanoma cell line, and generated BRS-3 knockout B16 cells (B16-KO) using CRISPR-Cas9 techniques [12]. A comparison of cell viability between H_2_O_2_-induced B16 cells and B16-KO cells (Figure 6D) revealed that BRS-3 may be involved in the cellular tolerance to hydrogen peroxide-induced injury. Additionally, Bantag-1 demonstrated efficacy in mitigating injury in B16 cells but not in B16-KO cells (Figure 6E), further supporting the role of BRS-3 in the process of cell injury.

Taken together, these findings indicated a prominent role of BRS-3 against oxidative stress-induced cell injury.

## 3. Discussion

In this study, we have identified a novel antagonist of the orphan receptor BRS-3. Our studies on BRS-3 overexpression in HEK293 and H1299 cells revealed that LIA was able to specifically inhibit MK-5046, a specific agonist of BRS-3, and induced calcium mobilization and IP1 accumulation with a sub-micromolar concentration range. To the best of our knowledge, LIA represents the first natural compound recognized as an antagonist for BRS-3.

Our team has been dedicated to the identification of exogenous ligands for GPCRs from natural products and their derivatives [11,12,14,15]. The screening of potential target compounds from natural products offers unique advantages. The vast diversity inherent in the structures of natural products is expected to significantly enhance the probability of identifying receptor ligands that possess high affinity and selectivity. Moreover, the well-documented pharmacological activities of natural product ligands facilitate the establishment of possible correlations with their corresponding receptors. This, in turn, provides invaluable insights and clues for exploring the unknown biological functions of receptors.

Despite numerous studies highlighting the important roles of BRS-3 in various physiological processes [5], thus far, no therapeutic agents have yet been developed for clinical use. This can largely be attributed to the limited research on BRS-3’s function and the lack of druggable ligands. MK-5046, for instance, has been demonstrated to cause cardiovascular complications, thereby restricting its clinical applicability [16]. Bantag-1, a high affinity and specific peptide antagonist of BRS-3, faces challenges in in vivo studies due to its short half-life [17]. ML-18, the first non-peptide BRS-3 antagonist, exhibited a moderate affinity for BRS-3, with an IC_50_ value of 4.8 μM, while maintain an affinity for GRPR (BB_2_), with an IC_50_ value of 16 μM [18]. The identification of the natural compound LIA as an antagonist of BRS-3 has significantly enhanced the feasibility of in vivo studies of BRS-3 and have improved its specificity. Given the known pharmacological effects, it may provide valuable insights into the novel function of BRS-3. Future work will focus on structural modifications of LIA to enhance its activity.

In our current study, we induced myocardial injury in vitro by treating cardiac cells with H_2_O_2_ and found that LIA exerted a protective effect on the injured cardiac cells. Furthermore, LIA was found to enhance the expression of the antioxidant enzymes SOD and CAT in H9c2 cells, while simultaneously inhibiting the production of lipid peroxidation end products, i.e., MDA and LDH. LIA also demonstrated protective properties in primary cardiomyocytes derived from rats and mice against H_2_O_2_-induced cell injury. Importantly, the protective effect of LIA was absent in primary cardiomyocytes from *Brs3*^−/y^ mice, strongly confirming that LIA acts by antagonizing BRS-3.

ILK is highly expressed in cardiac muscle, where it plays a key role in cell migration and the progression of cardiac diseases related to integrin function [19]. ILK binds to integrins and links integrins and receptor tyrosine kinases to the actin cytoskeleton, facilitating downstream signaling cascades, in particular the activation of AKT [20]. Additionally, the ERK/MAPK signaling pathway is recognized for its critical role in cardiac cell migration, survival, and cardiac repair [21]. Our proteomics studies illuminated the significance of ILK, AKT, and ERK in the protective mechanism of LIA against oxidative stress-induced cardiac injury. Subsequent immunoblotting analyses support that LIA reversed the H_2_O_2_-induced down-regulation of ILK, *p*-ERK1/2, and *p*-AKT (Ser^473^) in both H9c2 cells and primary rat cardiomyocytes. In H9c2 cells, all these beneficial effects of LIA were abolished when co-treated with the inhibitors targeting ILK, AKT, and ERK, respectively, further reinforcing the notion that LIA acts through these three pathways. Furthermore, ILK inhibitors reversed LIA-induced upregulation of *p*-AKT and *p*-ERK1/2, indicating that ILK is located upstream of AKT and ERK1/2. Taken together, by blocking BRS-3, LIA activates ILK, which in turn induces AKT and ERK1/2 phosphorylation, thereby exerting a protective effect against oxidative stress-induced cardiomyocyte injury.

The pharmacological profile of LIA points to the possibility that BRS-3 may play a crucial role in oxidative stress-induced cell injury, a previously unrecognized biological function of this orphan receptor. Therefore, to obtain further insight into the novel aspect of this receptor function, we utilized a variety of cell lines, as previously established in our research. HEK293 cells were stably transfected with murine-derived BRS-3 to facilitate high expression levels and functional studied in a well-characterized system. H1299 cells were stably transfected with human-derived BRS-3 to model the human receptor in a human cellular context, which is important for translational relevance. Additionally, B16 cells naturally expressed high levels of BRS-3 and were used to assess antagonizing effects in an endogenous receptor setting, mimicking physiological conditions. Our findings demonstrated that cell lines with elevated BRS-3 expression exhibited greater sensitivity to H_2_O_2_, rendering them more vulnerable to H_2_O_2_-induced cellular injury. Furthermore, Bantag-1, a well-known specific antagonist of BRS-3, also showed protective effects in both HEK293-BRS-3 and B16 cell lines exposed to H_2_O_2_. These results provide additional evidence supporting the involvement of BRS-3 in oxidative stress-induced cell injury.

## 4. Materials and Methods

### 4.1. Chemicals and Materials

LIA (cat#66056-19-7, purity ≥ 98%) was supplied by ChemFaces (Wuhan, China). MK-5046 was purchased from Shanghai Macklin Biochemical Technology Co., Ltd. (Shanghai, China). Bantag-1 was purchased from Sigma-Aldrich (St. Louis, MO, USA). OSU-T315, Capivasertib, FR 180,204 were purchased from MedChemExpress (Shanghai, China).

### 4.2. Cell Culture and Experiment Design

The H9c2 rat cardiomyocyte cell line was purchased from the Cell Bank of the Chinese Academy of Sciences (Shanghai, China) and cultivated in Dulbecco’s modified Eagle’s medium (DMEM, Gibco, Waltham, MA, USA) supplemented with 10% (*v/v*) fetal bovine serum (FBS, Gibco, Waltham, MA, USA) and 1% antibiotic penicillin/streptomycin solution (×100; Gibco, Waltham, MA, USA). Human embryonic kidney 293 mBRS-3 stable cell samples were kindly provided by Professor Olivier Civelli from the University of California, Irvine. All cells were placed in a humid incubator with 5% CO_2_ at 37 °C. B16 cells with BRS-3 knockout and H1299 stably expressing human BRS-3 cells were cultivated as described previously [8]. Primary cardiomyocytes were obtained by isolations from the ventricles of 1-day-old Wistar rats or C57BL/6J mice (SLAC Laboratory Animal Company, Shanghai, China). All animal experiments protocols were approved by the Animal Ethics Committee of SJTU (The registration number: O_A2021020-3).

Hydrogen peroxide (H_2_O_2_, Adamas, Shanghai, China) was used to induce oxidative injury in H9c2 cells. Cells were divided into four groups, namely the vehicle control group (Control), H_2_O_2_ treated group (H_2_O_2_), drug and H_2_O_2_-treated group (Drug + H_2_O_2_), and drug-treated group (Drug + Control). Cells were pretreated with tested compounds for one hour prior to exposure to H_2_O_2_. All experiments were repeated at least three times.

### 4.3. Calcium Mobilization

Intracellular Ca^2+^ mobilization was detected by fluorometric imaging plate reader (FLIPR) assay (Molecular Devices, Sunnyvale, CA, USA). HEK293-mBRS-3 stable cells were seeded into a black 96-well plate (Corning, Corning, NY, USA) with 8 × 10^4^ cells per well. For antagonist assays, antagonists were preincubated with the cells for 10 min before the addition of agonists. Calcium mobilization was monitored from the addition of the antagonist until 5 min after the addition of agonists. The maximum calcium response was determined by the peak calcium level elicited by the agonist. Normalization was performed by calculating the average calcium concentration over 20 s before agonist addition, which was set as the baseline (0%). The assay was conducted in a buffer solution containing 20 mM HEPES and 1× Hank’s balanced salt solution (HBSS) (Gibco, Waltham, MA, USA), pH 7.4, as the manual. The intracellular Ca^2+^ concentration was measured by FLIPR assay (Molecular Devices, Sunnyvale, CA, USA).

### 4.4. Dynamic Mass Redistribution (DMR) Assay

The DMR assay was performed using the Epic BT system (Corning, New York, NY, USA) as previously described [11]. HEK293-mBRS-3 stable cells were seeded into Epic 384-well biosensor microplates overnight; after 2 min baseline, compounds were added, and the DMR signals were recorded for 1 h. In the antagonistic assay, cells were incubated with LIA for 1 h before adding 25 nM of MK-5046, and then the DMR signals were monitored for 1 h.

### 4.5. Measurement of Inositol Phosphates (IP1) Accumulation

Following the IP-One-Gq KIT manufacturer’s protocol (Molecular Devices, Sunnyvale, CA, USA), IP1 accumulation was detected in H1299 and H1299-hBRS-3 stable cells based on the manufacturer’s protocol [22]. Briefly, cells were diluted in stimulation buffer at 5 × 10^5^ cells/mL in 384-well plates and were immediately treated with tested compounds for 1 h. Then, the IP1 d2 reagent (acceptor) was added to each well followed by the IP1 Tb cryptate antibody (donor), and the plate was sealed and incubated for another 1 h at room temperature. A Tecan Spark multimode microplate reader (Tecan, Männedorf, Switzerland) was used to determine the IP1 accumulation by using a homogeneous time-resolved fluorescence (HTRF) protocol (Ex 350 nm, Em 665/620 nm HTRF). Data were expressed as the percentage of the non-stimulation group.

### 4.6. Cell Viability Analysis

Cell viability was assessed by MTT assay. Cells were seeded into a 96-well plate at a density of 3 × 10^3^ cells per well, and the marginal wells were filled with PBS. Cells were incubated with various concentrations of LIA or Bantag-1 1 h before exposure to H_2_O_2_. For signaling pathway inhibitors, cells were pretreated 15 min before adding LIA or Bantag-1. Twenty-four hours after the H_2_O_2_ treatment, MTT solution (0.5 mg/mL, Sigma-Aldrich, St. Louis, MO, USA) was added to each well for another 4 h at 37 °C. After the incubation, the MTT reagent was removed and replaced with 100 μL of DMSO to dissolve the formazan for 15 min. Cell viability was analyzed by measuring the optical density at 570 nm with a microplate reader. All experiments were repeated in triplicate.

### 4.7. Malondialdehyde (MDA), Superoxide Dismutase (SOD), Lactate Dehydrogenase (LDH), and Catalase (CAT) Determination

The LDH level in the culture medium was tested using the LDH assay kit (Nanjing Jiancheng Bioengineering Institute, Nanjing, China). The intracellular MDA, SOD, and CAT activities were determined by using commercial kits (Nanjing Jiancheng Bioengineering Institute). Briefly, cells were lysed via the freeze–-thaw method and the reagents were mixed according to the manufacturer’s instructions. Protein concentrations were determined by using a Pierce™ BCA protein Assay kit (BCA, Thermo Scientific, Waltham, MA, USA).

### 4.8. Quantitative Real-Time Reverse Transcription Polymerase Chain Reaction (qRT-PCR)

Total RNA was extracted from cells by using TRIzol reagent (Beyotime, Shanghai, China), following the manufacturer’s instructions. Reverse transcription was performed by Rever Tra Ace^®^ qPCR RT Kit (Toroivd, Shanghai, China) and the fluorescence real-time PCR was performed using Bestar^®^ Sybr Green qPCR Master Mix (DBI^®^ Bioscience, Ludwigshafen, Germany). The sequences of the oligonucleotide primers are listed in Table 1. The parameter crossing point (Cp) values were normalized to those of glyceraldehyde 3-phosphate dehydrogenase (GAPDH), which was applied as the internal reference.

### 4.9. Proteomics Analysis by LC-MS/MS

H9c2 cells were seeded in 6-well plates at a density of 3 × 10^5^ per well. After 20 μM of LIA treatment for 1 h, cells were incubated for an additional hour with H_2_O_2_. The cells were harvested, and the proteins were extracted and measured by BCA. Then, cellular proteins (30 μg/each sample) were digested in trypsin (Promega, Madison, WI, USA) at 37 °C overnight. After desalting, digested peptides were dissolved in 10 μL of buffer A (0.1% formic acid in water), and equivalent peptides measured by Nanodrop were analyzed with EasynanoLC1000 coupled with a TripleTOF™ 5600+ system Mass spectrometer (AB Sciex, Framingham, MA, USA). Peptides were separated by a 150×0.3 mm reverse-phase column (ChromXP C18, 3 μm 120 Å, AB Sciex, Framingham, MA, USA). Buffer A and buffer B (80% acetonitrile with 0.1% formic acid) were applied as the mobile phases. The flow rate was 300 nL/min and the gradient was listed as follows: 2–20% buffer B (98 min), 20–30% buffer B (10 min), 30–95% buffer B (2 min), 95% buffer B (8 min), 95–2% buffer B (1 min), and kept in 2% buffer B for 1 min. The MS scan with a resolution of 70,000 ranged from 350 to 1500 m/z, and the scan for MS/MS ranged from 200 to 2000 m/z with a resolution of 17,500. MaxQuant (version 1.6.1.0, Martinsried, Germany) software was used for database search, and label-free quantification was performed using intensity-based absolute quantification (iBAQ). Differently expressed proteins were screened following the criteria of at least two unique peptides, 1.5-fold change (FC), and *p* value < 0.05.

### 4.10. Western Blot Assay

Protein preparation was the same as described in Section 4.8, and cells were pretreated with signaling inhibitors 15 min before the addition of LIA or Bantag-1. After 10 min of denaturing at 99 °C, proteins were loaded and separated by sodium dodecyl sulfate–polyacrylamide gel electrophoresis (SDS-PAGE), then transferred to nitrocellulose membranes. The membranes were blocked for 1 h in 5% BSA dissolved in Tris-buffered saline with 0.1% Tween (TBST) and incubated with primary antibodies with a dilution of 1:1000. The primary antibodies targeting phospho (*p*)-AKT, *p*-ERK, *p*-P70S6K, and β-Tubulin were purchased from cell signaling technology (Danvers, MA, USA), while the anti-ILK was purchased from Servicebio^®^ (Wuhan, China). The membranes were washed with TBST, incubated with secondary antibodies (Teyebio, Shanghai, China) for 1 h, then detected with a Tanon 5200 multi-imaging system (Tanon, Shanghai, China).

### 4.11. Molecular Docking

The predicted 3D structure of human BRS-3 was downloaded from the AlphaFold Protein Structure Database (P32247). The human BRS-3 structure was prepared in Protein Preparation Wizard of Maestro (Schrödinger LLC, New York, NY, USA): hydrogen atoms were added; bond orders were assigned; H-bond assignment was optimized at pH 7.0 using PROPKA. All atoms were energy-minimized to reach the convergent RMSD of 0.3 Å with the OPLS4 force field. The three-dimensional conformation of LIA was prepared in LigPrep with the OPLS4 force field. The Induced Fit Docking module was used to fine-tune the receptor structure. The centroid of residues Arg^127^, His^294^, and His^107^ was defined as the box center. Then, a protein grid box for docking was generated by enclosing the residues in the box at a size of 10 Å × 10 Å × 10 Å centered on the LIA using the receptor grid generation module with no constraints. To probe the possible binding mode of LIA with the BRS-3 prepared structure, we conducted molecular docking using the Extra Precision (XP) Glide module. The docking parameters were set to default. Molecular mechanics/generalized Born and surface area solvation (MM-GBSA) were calculated using the Prime module.

### 4.12. Statistical Analysis

All values were displayed as mean ± standard deviation (SD), and the statistical analysis was performed by using GraphPad Prism 8.0 software. Statistical difference was analyzed by one-way analysis of variance followed by the Dunnett post hoc test. The difference was significant if *p* < 0.05.

## 5. Conclusions

The present study elucidates that the natural compound LIA serves as an exogenous antagonist of BRS-3. LIA was effective in preventing H_2_O_2_-induced cell injury in both H9c2 cells and primary cardiomyocytes. This protective effect was associated with the activation of integrin/ILK/AKT and ERK/MAPK signaling pathways. This study also contributes significantly to expanding the understanding of the potential roles of BRS-3, uncovering previously unknown pharmacological functionality of this orphan receptor. These findings provide promising insights into potential therapeutic interventions for cardiac injuries and may pave the way for future drug development in this area.

## Figures and Tables

**Table 1:** Primer sequences for qRT-PCR.

*Brs3*	Forward	5′-GAAACATCAAGCTCTGCCGTCT-3′
	Reverse	5′-CCACTGAAATGATCACAGCAT-3′
*Actb*	Forward	5′-CGAGTACAACCTTCTTGCAGC-3′
	Reverse	5′-TATCGTCATCCATGGCGAACTG-3′

## Data Availability

All datasets generated or analyzed during this study are available from the corresponding author on reasonable request.

## Supplementary Materials

The following supporting information can be downloaded at: https://www.mdpi.com/article/10.3390/ijms26062745/s1.

## Abbreviations

The following abbreviations are used in this manuscript:

BRS-3/BB_3_	Bombesin receptor subtype-3
DMR	Dynamic mass redistribution
FLIPR	Fluorometric imaging plate reader
GPCR	G protein-coupled receptor
GRPR/BB_2_	Gastrin-releasing peptide receptor
H_2_O_2_	Hydrogen peroxide
HTRF	Homogeneous time-resolved fluorescence
iBAQ	Intensity-based absolute quantification
ILK	Integrin-linked kinase
LIA	Licoisoflavone A
MAPK	Mitogen-activated protein kinases
NMBR/BB_1_	Neuromedin B receptor
