# Supplementary material for: Identification of a Novel Antagonist of BRS-3 from Natural Products and Its Protective Effects Against H2O2-Induced Cardiomyocyte Injury
Source: Int J Mol Sci. 2025 Mar 18;26(6):2745. doi: 10.3390/ijms26062745 (PMC11943355; doi:10.3390/ijms26062745)
Supplement: Supplementary file 1 [file ijms-26-02745-s001.zip › ijms-3504425-supplementary.pdf]

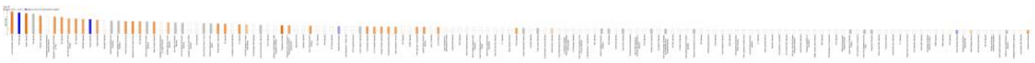

Fig. S1. IPA analyzed the canonical pathway enrichment.

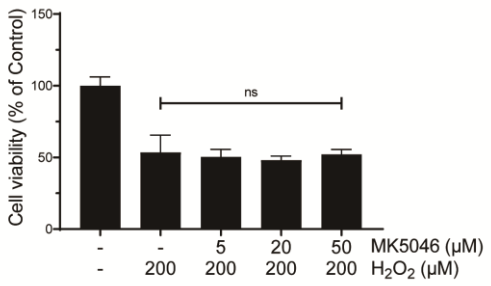

Fig. S2. The effect of MK-5046, an agonist of BRS-3 on the cell viability in H_2O_2-induced H9c2 cell injury. The values are represented as means ± SD, n=6.

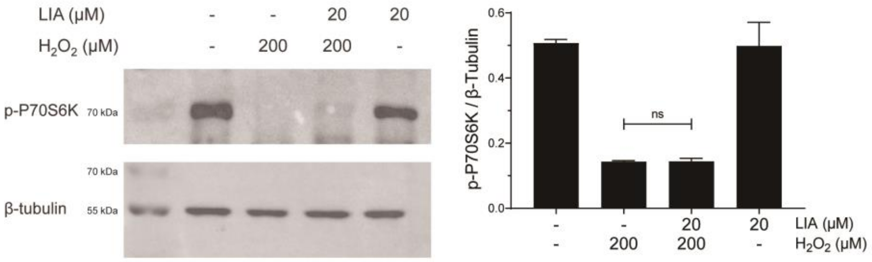

Fig. S3. The effect of LIA on the mTOR signaling pathway in H9c2 cells. The values are represented as means ± SD, n=3.

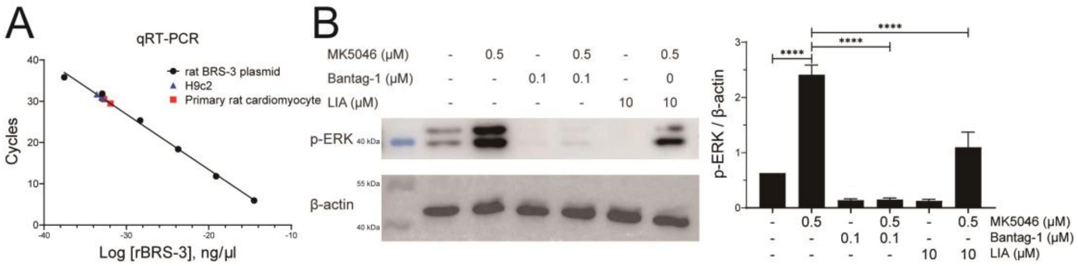

Fig. S4. The expression and function of BRS-3 in rat cardiomyocytes. The mRNA expression of BRS-3 in H9c2 cells and primary rat cardiomyocytes (A). The effect of the agonist and the antagonist of BRS-3 on ERK phosphorylation in H9c2 cells (B). The values are represented as means ± SD, n=3. ****$p < 0.001$.
